# Effects of Electron Beam Irradiation on Zearalenone and Ochratoxin A in Naturally Contaminated Corn and Corn Quality Parameters

**DOI:** 10.3390/toxins9030084

**Published:** 2017-02-27

**Authors:** Xiaohu Luo, Lijun Qi, Yuntao Liu, Ren Wang, Dan Yang, Ke Li, Li Wang, Yanan Li, Yuwei Zhang, Zhengxing Chen

**Affiliations:** 1 State Key Laboratory of Food Science and Technology, National Engineering Laboratory for Cereal Fermentation Technology, School of Food Science and Technology, Jiangnan University, Wuxi 214122, China; xh06326@gmail.com (X.L.); qlj_jiangnan@163.com (L.Q.); nedved_wr@jiangnan.edu.cn (R.W.); m18811990310_2@163.com (D.Y.); 6160112134@vip.jiangnan.edu.cn (K.L.); legend0318@hotmail.com (L.W.); lyn19881012ph@163.com (Y.L.); 2 College of Food Science, Sichuan Agricultural University, Yaan 625014, China; ytliu123456@foxmail.com; 3 EL PONT Radiation Technology Co., Ltd., Wuxi 214151, China; yuwei.zhang@elpont.net

**Keywords:** zearalenone, ochratoxin A, electron beam irradiation, degradation, quality

## Abstract

Zearalenone (ZEN) and ochratoxin A (OTA) are secondary toxic metabolites widely present in grains and grain products. In this study, the effects of electron beam irradiation (EBI) on ZEN and OTA in corn and the quality of irradiated corn were investigated. Results indicated that EBI significantly affected ZEN and OTA. The degradation rates of ZEN and OTA at 10 kGy in solution were 65.6% and 75.2%, respectively. The initial amounts significantly affected the degradation rate. ZEN and OTA in corn were decreased by the irradiation dose, and their degradation rates at 50 kGy were 71.1% and 67.9%, respectively. ZEN and OTA were more easily degraded in corn kernel than in corn flour. Moisture content (MC) played a vital role in ZEN and OTA degradation. High MC was attributed to high ZEN and OTA degradation. The quality of irradiated corn was evaluated on the basis of irradiation dose. L* value changed, but this change was not significant (*p* > 0.05). By contrast, a* and b* decreased significantly (*p* < 0.05) with irradiation dose. The fatty acid value increased significantly. The pasting properties, including peak, trough, breakdown, and final and setback viscosities, were also reduced significantly (*p* < 0.05) by irradiation. Our study verified that EBI could effectively degrade ZEN and OTA in corn. Irradiation could also affect corn quality.

## 1. Introduction

The safety of agricultural products, such as cereals, oilseeds, and other crops, is of considerable concern worldwide, and their contamination with mycotoxins is a significant problem [1]. Mycotoxins are secondary toxic metabolites mainly from the mycelial structure of filamentous fungi that grow naturally on agricultural products and produce toxins under favorable conditions. Mycotoxins are accounted for the losses of millions of dollars in infected agricultural products and human and animal health annually worldwide. To date, over 400 mycotoxins and fungal metabolites have been reported; among these mycotoxins, zearalenone (ZEN) and ochratoxin A (OTA) are two of the most toxic and widespread compounds with remarkable agro-economic importance [2].

ZEN is an estrogenic compound primarily produced by *Fusarium* species [3] and found in cereals, including corn, barley, oats, wheat, sorghum, millet, and rice. Among these cereals, corn is the most frequently contaminated with ZEN [4]. In animals, ZEN mainly affects the reproductive system because of its estrogenic effect; ZEN and its derivatives also severely influence fertility in males and females [5]. Pigs have been shown to be the most sensitive animals to ZEN; when exposed to ZEN, sexually mature gilts experience swelling of the vulva and mammary glands and occasional vaginal and rectal prolapses [6].

OTA is mainly produced by *Aspergillus ochraceus* and *Penicillium verrucosum* in a wide range of foodstuff, such as cereals, coffee, beer, dried fruits, spices, fruit juice, milk, and wines [7,8,9,10]. With the wide existence of the toxin in foodstuff, it has also been detected in human blood serum, breast milk, and kidney [11]. OTA can elicit toxic effects on animal species, and its main target organ is the kidney [12]. OTA can induce porcine nephropathy in pigs and Balkan endemic nephropathy in humans [13,14]. OTA is also classified as a possible human carcinogen (Group 2B) by the International Agency for Research on Cancer [15].

Several factors, such as storage and environmental and ecological conditions, can influence the presence of mycotoxins in agricultural products, but most of these factors are uncontrollable by humans. The majority of mycotoxins also demonstrate remarkable stability. As such, mycotoxin contamination persists in the food industry despite improvements in food handling, storage, and processing; therefore, detoxification approaches should be further developed. Physical, chemical, and biological methods can be used to detoxify mycotoxins in foodstuff [16,17,18,19,20]. However, most of these methods cause nutrient loss, alter sensory attributes, and produce chemical residues, leading to limited applications. Thus, effective and practical methods should be established for mycotoxin detoxification.

Food irradiation is a physical method that promotes food safety and has thus been gradually applied in the food industry. Since 1981, an overall dose of up to 10 kGy of food irradiation has been considered safe and effective [21]. In recent years, doses above 10 kGy have also been considered safe for several niche products and markets [22]. The application of radiation of up to 50 kGy in contaminated animal feeds has been approved by the FDA [23]. Irradiation can react with molecules in organisms and thus induce physical, chemical, and biological effects, which cause injury or death of a living body. Through this approach, the microbiological safety of food can be improved, and its shelf life can be prolonged without substantial alteration [24,25]. Irradiation can also eliminate pests on agricultural commodities; consequently, food losses are minimized and use of chemical fumigants and additives is avoided [26]. The method can reduce the amounts of mycotoxins, such as AFB_1_, ZEN, and OTA [27]. Irradiation severely affects mold viability and inhibits fungal development and mycotoxin production in commodities; ionizing radiation can also directly act on mycotoxins under specific conditions [27]. Corn, as one of the most important crops worldwide, can be used as food ingredients, feedstuff, and industrial materials, but this crop has been contaminated by mycotoxins, including ZEN and OTA [28]. These mycotoxins cause grain wastage, resulting in economic losses and increased health risk among humans and animals. To eliminate these mycotoxins, agricultural researchers apply irradiation, such as gamma irradiation, which is frequently used in the food industry because of its penetration efficiency. Electron beam irradiation (EBI) is also applied and is more convenient, less costly, and safer than gamma irradiation [29]. In the present research, the effects of EBI on ZEN and OTA in naturally co-contaminated corn were investigated.

Although mycotoxins have been detoxified through irradiation, the effects of detoxification remain controversial. The simultaneous detoxification of ZEN and OTA in naturally contaminated corn by EBI has yet to be reported. Our study is essential because different irradiation doses, sample appearances, and mycotoxins may elicit various detoxification effects. This study aimed to (1) investigate the effects of EBI on ZEN and OTA standard solutions; (2) evaluate the detoxification effects of EBI on corn; and (3) assess the quality parameters of irradiated corn.

## 2. Results and Discussion

### 2.1. Effects of EBI on ZEN and OTA Solutions

**Figure 1:**
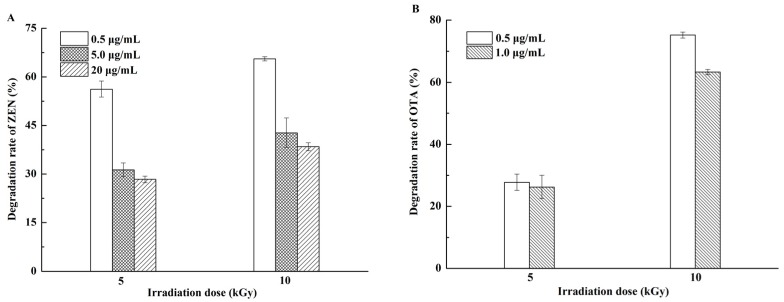
Degradation rates of mycotoxin solutions by electron beam irradiation (EBI). (**A**) Zearalenone (ZEN) solutions of 0.5, 5.0, and 20.0 μg/mL were exposed to 5 and 10 kGy; (**B**) ochratoxin A (OTA) solutions of 0.5 and 1.0 μg/mL were exposed to 5 and 10 kGy.

Figure 1A,B show that the degradation rates of ZEN and OTA solutions increased with the irradiation dose. At the EBI dose of 10 kGy, the degradation rates of ZEN at 0.5, 5, and 20 μg/mL were 65.6%, 42.8%, and 38.4%, respectively. OTA at 0.5 and 1.0 μg/mL decreased by 75.2% and 63.2% at the same EBI dose, respectively. This trend was consistent with the findings of Peng et al., who revealed that EBI at 2.5 kGy destroyed 92.88%, 7.20%, and 30.83% OTA in water, acetonitrile, and methanol–water (60:40, *v*/*v*), respectively. At 10 kGy, the corresponding degradation rates increased to 99.34%, 68.76%, and 66.75%. The degradation rate of OTA in water was significantly higher than that in organic solvents [30]. EBI causes the splitting of water molecules into several radiolysis products, including *e*_aq_-, H•, and HO• [31], which are reactive to OTA. However, the degradation rate of OTA in acetonitrile in our study was higher than that reported by Peng et al. [30] probably because of different treatment conditions, such as volume, container, and manner of placement.

The results also indicated that the initial amount of mycotoxins negatively influenced degradation. In particular, at 20.0 μg/mL ZEN solution, the degradation rate was merely 38.4% at the highest dose. The amount of free radicals produced by EBI was extremely small in organic solvents [30]. The decrease in ZEN and OTA was mainly ascribed to the direct degradation of ZEN and OTA molecules because the splitting of ZEN and OTA molecules caused by EBI was roughly similar under the same irradiation dose. Therefore, ZEN and OTA solutions with low concentration presented a high rate of decline. Our result was consistent with findings of previous reports. Van Dyck et al. [32] found that increasing the dose of gamma radiation can destroy the increase in aflatoxin B_1_ (AFB_1_), but the effect of gamma rays is substantially reduced when the concentration of AFB_1_ is increased 50-fold. Liu et al. [33] also showed that the AFB_1_ degradation rate decreases in the following order: 5 ppm > 1 ppm > 0.5 ppm, exhibiting marked initial concentration-dependent phenomenon in aqueous medium.

### 2.2. Effect of EBI on Naturally Contaminated Corn

**Figure 2:**
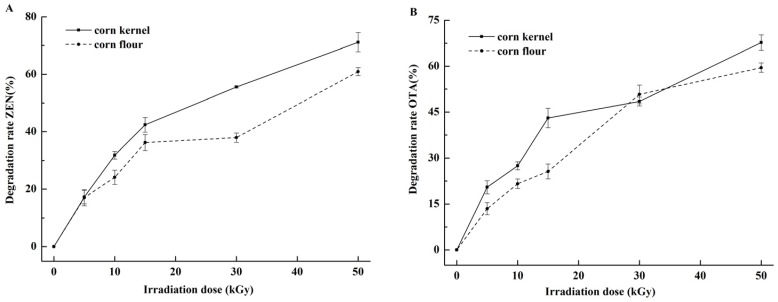
Degradation rates of ZEN and OTA in corn samples by EBI. (**A**) ZEN in naturally contaminated corn kernel and corn flour after irradiation from 5 kGy to 50 kGy; (**B**) OTA in naturally contaminated corn kernel and corn flour after irradiation from 5 kGy to 50 kGy.

EBI-induced ZEN and OTA detoxification in contaminated corn kernel or corn flour is shown in Figure 2A,B. The initial contents of ZEN and OTA were 2812.5 and 60.18 μg/kg, respectively. The degradation rates increased with the irradiation dose. At the irradiation dose of 50 kGy, the degradation rates of ZEN and OTA in corn kernel were 71.1% and 67.9%, respectively. Stepanik et al. [34] found that the reduction of deoxynivalenol level in wet distillery grain, distiller’s solubles, and stillage exhibits a dose-dependent increase. Aziz et al. [35] reported that gamma irradiation is effective against AFB_1_, ZEN, and OTA in food and agricultural products; at 20 kGy, AFB_1_ and ZEN are completely disrupted and OTA content in samples is reduced by 72%–76%. The differences in the degradation rates from the current findings may be ascribed to the amounts of ZEN and OTA present in the samples before irradiation [36] and the presence of water [37]. The initial content negatively affects the degradation of mycotoxins. The corn samples in our study were intensely contaminated with ZEN and OTA, and the initial positive moisture content (MC) in degradation was extremely low (11.9%). The penetration ability of EBI is more limited than that of gamma irradiation [38].

Figure 2A,B reveal that the degradation rates of ZEN and OTA in corn kernel were higher than those in corn flour, probably because ZEN and OTA were mainly distributed to the surface of corn [39], which was accessible for irradiation of kernels. Yin et al. [40] found that complete corn granules improve the degradation effect of ZEN when irradiated by gamma rays.

### 2.3. Effect of MC of Corn Kernel on EBI

**Figure 3:**
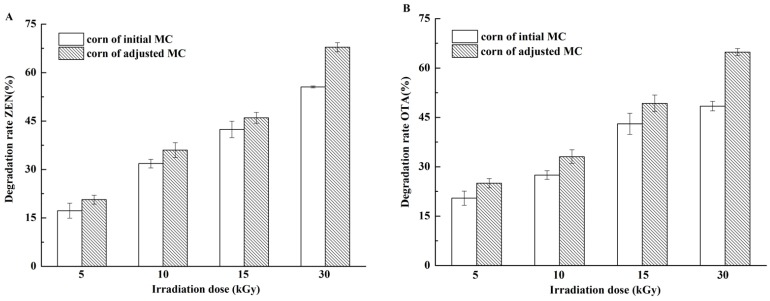
Degradation rates of ZEN and OTA in naturally contaminated corn kernel with different moisture content (MC) levels. (**A**) ZEN in naturally contaminated corn kernel after irradiation from 5 kGy to 30 kGy; (**B**) OTA in naturally contaminated corn kernel after irradiation from 5 kGy to 30 kGy.

The different MCs of corn kernels at various irradiation doses are shown in Figure 3. The results indicated that increased MC was favorable for EBI to destroy increasing ZEN and OTA contents in corn kernels. Radiolysis can be generated in water and thus plays a vital role in the reaction with organic compounds. We speculated that more free radicals are generated at higher MC and consequently induce violent interactions [41]. Frank et al. [42] reported that dried aflatoxins are difficult to be degraded, whereas the amounts of aflatoxins in phosphate solution decrease by approximately 90% at the irradiation doses of 1 and 2.5 kGy. Jalili et al. [37] found that MC shows positive significant effects (*p* < 0.05) on mycotoxin reduction; in spite of the gamma irradiation dose and type of mycotoxin, maximum reduction values of 45.87% and 55.27% were found for MC of 12% and 18%, respectively. These reports were consistent with our findings, that is, high MC was favorable for the effect of irradiation on mycotoxins.

### 2.4. Color of Irradiated Corn Kernel and Flour

Table 1 describes the changes in the color of irradiated corn at different doses. L*, a*, and b* values represent whiteness, redness, and yellowness, respectively. L* showed insignificant changes with the irradiation dose (*p* > 0.05), whereas a* and b* decreased significantly (*p* < 0.05). Naseer et al. [43] reported that the L* and b* values of whole wheat flour decrease with gamma irradiation dose, but the variation is not significant. In addition, the a* value increased with the irradiation dose. Abu et al. [44] observed a decrease in L* for cowpea seeds upon irradiation. Wani et al. [45] observed an increase in a* values of arrowhead tuber starch and flour at the irradiation dose from 0 kGy to 10 kGy. The differences in results were probably attributed to various matrices. Corn shows abundant variation in health-promoting carotenoids, including lutein and zeaxanthin [46], which provide the color of corn. EBI can induce the splitting of these molecules, resulting in the decrease in a* and b* values. In addition, the decrease in a* and b* in corn flour was sharper than that in corn kernel. This result may occur because the pigments in flour were more exposed to EBI.

### 2.5. Free Fatty Acid Value of Irradiated Corn Kernel and Flour

**Figure 4:**
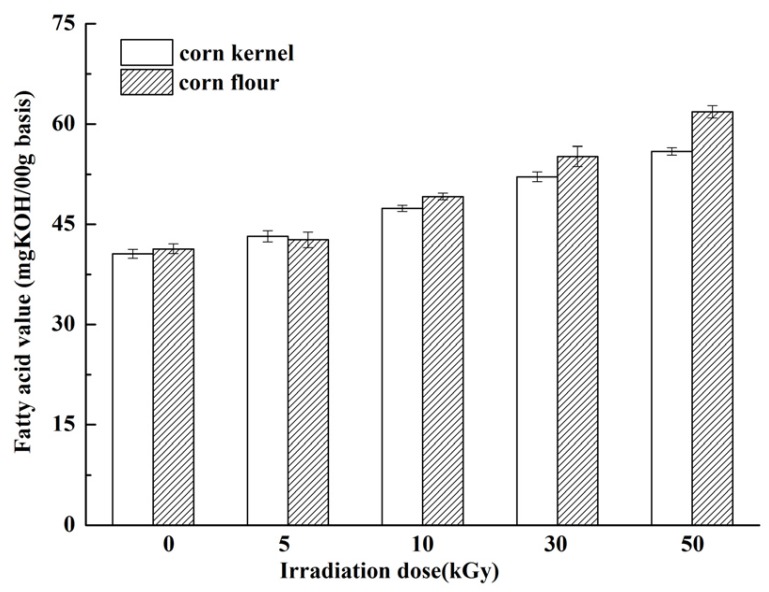
Free fatty acid values of irradiated corn kernel and corn flour after irradiation from 5 kGy to 50 kGy.

Irradiation can promote the oxidation of fats, forming free radicals and leading to rancidity and odor or color changes. High-dose irradiation may decompose the fat components, thereby increasing the free fatty acid values. Free fatty acid value is a significant indicator used to measure the changes in the quality of corn during storage [47]. Figure 4 shows the changes in the free fatty acid values of irradiated corn kernel and flour. The free fatty acid value increased as irradiation dose increased. At the highest dose, the fatty acid values were 55.9 mg KOH/100 g and 61.82 mg KOH/100 g for corn kernel and corn flour, respectively, indicating that corn was still normal (≤65 mg KOH/100 g) [47]. These results indicated that low doses did not change the free fatty acid value considerably and probably occurred because fat components were protected by corn husks. The energy produced from EBI weakened upon reaching the internal amount. This mechanism also explains why the free fatty acid value changed more evidently in corn flour than in corn kernel, because fats were exposed to the surface after grinding. Marathe et al. [48] found that irradiation treatment up to 10 kGy dose does not change the free fatty acid values in red kidney bean because of the low fat content. In addition, the irradiation dose was considerably higher in the present study.

### 2.6. Pasting Properties of Irradiated Corn Kernel and Flour

As shown in Table 2, the pasting properties, including peak, trough, breakdown, and final and setback viscosities, of corn kernel and corn flour were decreased significantly (*p* < 0.05) by irradiation. At 5 and 10 kGy, the peak and trough viscosities of corn kernel decreased less sharply than those of corn flour probably because of the protective effect of corn husk. At 30 and 50 kGy, the ranges of decrease in these parameters were similar. This phenomenon occurred possibly because energy was sufficient as irradiation dose increased regardless of the matrix form. Irradiation-induced decreases in pasting properties have also been reported in rice starch [49], maize, and bean flour [50]. The high reduction in starch viscosity is believed to be due to the degradation of starch because of irradiation. De Kerf et al. [51] found a marked reduction in the amylopectin fraction of various starches with irradiation. Amylopectin presents a negative correlation with starch viscosities. Joseph et al. [52] analyzed the sizes of cowpea starch granules, because granule size partly contributes to starch pasting and retrogradation properties. However, their results indicated that irradiation apparently does not cause fissures or splitting in cowpea starch granules. Sokhey et al. [53] employed scanning electronic microscopy and found that irradiation at 31 kGy does not cause physical damage to waxy maize starch granules. Damage to starch granules by irradiation dose appears in the form of changes in the structure of starch molecules. Decreases in pasting properties, such as breakdown and setback values may help ease cooking and reduce starch retrogradation, respectively [54]. However, such changes are undesirable at high irradiation doses.

## 3. Conclusions

EBI was confirmed as an effective method to degrade ZEN and OTA in corn kernel and corn flour. The degradation rate increased as irradiation dose increased. High MC was favorable for the degradation effect, and the quality parameters of corn were affected by irradiation. a* and b* decreased as irradiation dose increased, and the fatty acid value increased. The pasting properties, including peak, trough, breakdown, final, and setback viscosities, were reduced significantly (*p* < 0.05) by irradiation. However, other factors, such as temperature, pH, and grain thickness, favorable for optimum EBI should be investigated. Further studies should be performed to determine the degradation products and toxicities of ZEN and OTA following EBI.

## 4. Materials and Methods

### 4.1. Materials

Corn samples naturally co-contaminated with ZEN and OTA were obtained from Taixing City (Jiangsu Province, China) and stored at 4 °C before treatment was administered. ZEN and OTA in the form of dust (purity ≥ 98%) were purchased from J&K Scientific Ltd. (Shanghai, China). Ultrapure water (resistivity ≥ 18 MΩ/cm) was obtained from a Millipore-Q SP Reagent Water system (Millipore, Bedford, MA, USA) and pre-filtered through a 0.22 µm filter. High-performance liquid chromatography (HPLC)-grade methanol was supplied by Fisher Scientific (Pittsburgh, PA, USA). All other analytical grade chemicals or reagents were supplied by J&K Scientific Ltd. (Shanghai, China).

### 4.2. Instruments

Samples were irradiated at doses of 5–50 kGy using an industrial electron accelerator at room temperature (25 °C) in Wuxi EL PONT Radiation Technology Co., Ltd., China. The energy of accelerated electrons was 5 MeV, and the beam current was 20 mA with 1000 mm scan width and a dose rate of 2 kGy/s. An Agilent 1260 series HPLC system (Agilent Technologies, Palo Alto, CA, USA) with a fluorescence detector was used for mycotoxin analysis. An Agilent ZORBAX SB-C18 chromatographic column (150 mm × 4.6 mm; 5 µm particle size; Agilent Technologies) was utilized for separation.

### 4.3. EBI of ZEN and OTA Standards

ZEN solutions at different concentrations (0.5, 5.0, and 20.0 μg/mL) were prepared in methanol, and OTA solutions (0.5 and 1.0 μg/mL) were prepared in acetonitrile. The above solutions (1 mL) were added into 5 mL polyethylene centrifuge tubes and irradiated at 5 and 10 kGy flatwise. After irradiation, the degradation rates of ZEN and OTA were analyzed by HPLC (Agilent Technologies, Palo Alto, CA, USA).

### 4.4. EBI of Contaminated Corn

Corn kernels (200 g) were placed in a polyethylene bag and spread to a thickness of 1 cm. Corn kernel was also ground by using a swing pulverizer and passed through mesh sieves with 0.9 mm diameter. Corn flour (200 g) was treated under the same condition. Corn kernels and corn flour were irradiated at doses of 0, 5, 10, 30, and 50 kGy. After irradiation, all samples were stored at 4 °C before HPLC analysis. The degradation rate coefficient is defined as mycotoxin contents in the irradiated corn sample versus mycotoxin contents in the unirradiated corn sample.

### 4.5. Preparation of ZEN and OTA Extracts

ZEN and OTA in the corn samples were extracted using previously described methods [55]. Corn kernels were ground and then sifted through 30-mesh sieves. Corn flour (25 g) was placed in a 250 mL flask, and 5 g of sodium chloride and 100 mL of acetonitrile–water extraction solvent (90:10 *v*/*v*) were added. The flask was shaken for 45 min at 200 rpm, and the extract was filtered through a pre-folded filter paper to obtain crude filtrates. Afterward, 4 mL of crude filtrate was passed through a Bond Elut Mycotoxin clean-up column (12165001B, 45 mm/1000 mg; Agilent), and 2 mL of the extract was collected for ZEN analysis. Then, 2 mL of the crude filtrate was obtained directly for OTA analysis. Both extracts were dried under a nitrogen stream at 50 °C, reconstituted with 1 mL of the HPLC mobile phase, and vibrated on a vortex blender for 40 s. Finally, 1 mL of the supernatant was passed through an organic membrane filter (0.22 μm) and collected with a 2 mL calibrated glass vial. The sample was stored at 4 °C before HPLC analysis was performed.

### 4.6. HPLC Conditions

The HPLC conditions were based on previously described methods [55]. The mobile phase for ZEN analysis was a methanol–water solution (60:40, *v*/*v*) with an injection volume of 20 μL and flow rate of 1 mL/min. The excitation wavelength of the fluorescence detection detector was 274 nm, and the emission wavelength was 440 nm. For OTA analysis, acetonitrile–water–acetic acid (56:43:1, *v*/*v*/*v*) constituted the mobile phase with an injection volume of 20 μL and a flow rate of 0.9 mL/min. The excitation and emission wavelengths were 333 and 477 nm, respectively. The detection limits of ZEN and OTA were 5.0 and 1.0 μg/kg, respectively.

### 4.7. MC Determination

MC was determined in accordance with the Chinese National Standard GB/T 5009.3-2010 [56]. The initial MC of corn was 11.9% and adjusted to 16.7% by adding artificial water to meet the experiment requirement. Corn samples were left with water overnight (10 h) before irradiation treatment.

### 4.8. Color Determination of Corn Flour

The color of corn flour was evaluated using Ultra Scan Pro 1166 (HunterLab, Reston, VA, USA) and expressed as L*, a*, and b* values, which represent the units of whiteness, redness, and yellowness, respectively.

### 4.9. Determination of Fatty Acid Values

Fatty acid values were determined in accordance with the Chinese National Standard GB/T 29405-2012 [57]. Ground corn sample (10.00 g) was extracted by using 50 mL of anhydrous ethanol for 30 min on an oscillator. Filtrates (25 mL) were collected in a measuring cup, and 50 mL of carbon dioxide-free distilled water was added. A potential titrator was used to record the volumes of KOH standard titration solution. The final results were expressed in terms of milligrams of KOH required to neutralize free fatty acids in 100 g of corn flour (dry basis).

### 4.10. Pasting Property Determination of Corn Flour

The pasting properties of corn flour were determined in accordance with the Chinese National Standard GB/T 24853-2010 [58] by using a RapidVisco-Analyzer (RVA 4500, Pertone Corporation, Australia). The standard profile 1 of the RVA (RapidVisco-Analyzer) was employed. The process of this profile is in the following: the samples (3 g) in 25 mL of distilled water were heated from an initial temperature of 50 °C to 95 °C in 13 min, held at this temperature for 4 min, and cooled to 50 °C for 13 min.

### 4.11. Statistical Analysis

Values were expressed as standard deviations (SDs) of means. Data were evaluated through ANOVA in SPSS 16.0 (SPSS Inc., Chicago, IL, USA). One-way ANOVA was performed to verify the significant differences between irradiation doses and corn quality. Results were considered significant when *p* ≤ 0.05.

## Figures and Tables

**Table 1:** L*, a*, and b* values of irradiated corn kernel and corn flour after irradiation from 5 kGy to 50 kGy.

Sample	Irradiation Dose/kGy	L*	a*	b*
Corn kernel	0	89.23 ± 0.72 ^aa’^	2.82 ± 0.01 ^aa’^	21.75 ± 0.22 ^aa’^
	5	89.25 ± 0.09 ^a^	2.46 ± 0.11 ^b^	20.44 ± 0.03 ^b^
	10	89.90 ± 0.13 ^a^	2.12 ± 0.08 ^c^	20.40 ± 0.08 ^b^
	30	90.12 ± 0.50 ^a^	2.08 ± 0.01 ^d^	19.42 ± 0.16 ^b^
	50	89.94 ± 0.05 ^a^	2.17 ± 0.02 ^c^	18.70 ± 0.01 ^c^
Corn flour	5	88.73 ± 0.64 ^a’^	2.60 ± 0.01 ^b’^	21.24 ± 0.20 ^b’^
	10	88.76 ± 0.42 ^a’^	2.51 ± 0.02 ^c’^	20.57 ± 0.42 ^c’^
	30	89.37 ± 0.51 ^a’^	1.81 ± 0.02 ^d’^	18.70 ± 0.04 ^d’^
	50	90.00 ± 0.61 ^a’^	1.57 ± 0.01 ^e’^	17.94 ± 0.05 ^e’^

Sample at 0 kGy was used as control. The letter a was used as comparison for corn kernel; the letter a’ was used as comparison for corn flour. Different letters in the same row suggest significant inter-group differences, *p* < 0.05; the same letters in the same row suggest insignificant inter-group differences, *p* > 0.05.

**Table 2:** Pasting properties of irradiated corn kernel and corn flour after irradiation from 5 kGy to 50 kGy.

Sample	Irradiation Dose (kGy)	Peak Viscosity (cp)	Trough Viscosity (cp)	Breakdown Viscosity (cp)	Final Viscosity (cp)	Setback Viscosity (cp)
Corn kernel	0	1290 ± 35 ^aa’^	673 ± 22 ^aa’^	617 ± 32 ^aa’^	1939 ± 34 ^aa’^	1266 ± 34 ^aa’^
	5	1026 ± 28 ^b^	303 ± 21 ^b^	723 ± 46 ^b^	592 ± 41 ^b^	289 ± 36 ^b^
	10	352 ± 21 ^c^	72 ± 3 ^c^	281 ± 3 ^c^	117 ± 4 ^c^	46 ± 3 ^c^
	30	77 ± 2 ^d^	35 ± 1 ^d^	42 ± 3 ^d^	57 ± 1 ^d^	22 ± 1 ^d^
	50	25 ± 1 ^e^	12 ± 2 ^e^	13 ± 1 ^e^	24 ± 1 ^e^	12 ± 1 ^e^
Corn flour	5	728 ± 38 ^b’^	222 ± 21 ^b’^	506 ± 43 ^b’^	668 ± 27 ^b’^	446 ± 42 ^b’^
	10	278 ± 46 ^c’^	58 ± 2 ^c’^	220 ± 22 ^c’^	135 ± 25 ^c’^	77 ± 12 ^c’^
	30	101 ± 4 ^d’^	34 ± 4 ^d’^	67 ± 1 ^d’^	52 ± 1 ^d’^	18 ± 1 ^d’^
	50	35 ± 2 ^e’^	18 ± 3 ^e’^	17 ± 2 ^e’^	28 ± 1 ^e’^	10 ± 1 ^e’^

Pasting properties at 0 kGy was used as control. The letter a was used as comparison for corn kernel; the letter a’ was used as comparison for corn flour. Different letters in the same row suggest significant inter-group differences, *p* < 0.05; the same letters in the same row suggest insignificant inter-group differences, *p* > 0.05.
